# Poisoned Wounds

**Published:** 1828-10

**Authors:** 


17. *Poisoned wounds. Cupping Glasses. Pressure. Ligatures.* In the second number of our Journal, we gave a short analysis of the experiments of Dr. Pennock on the efficacy of pressure in poisoned wounds. In the periodical work just quoted, Dr. Aristide Rodrique has published a series of analogous experiments, performed about the same time by himself. His experiments were made on cats and rabbits, with strichnia, and hydrocyanic acid of the strongest kind. The results were found to coincide with those of Dr. Pennock, and give great support to the opinion, that it is not a *vacuum over the wound*, as Dr. Barry supposed, but *pressure around it*, that preserves the constitution from injury;—and that this pressure, (which in every case may be promptly and successfully made, without the aid of a cupping glass or a physician) is likely to be successful is extremely probable. The following are his conclusions:

"1st. A vacuum caused over a part under which a poison has been deposited, will prevent such a poison from producing its usual effects so long as this vacuum exists.

"2d. All constitutional symptoms immediately disappear on the establishment of a vacuum over the poisoned surface, and by removing the poison by an incision, the life of the animal may be saved, provided too great a quantity be not previously absorbed.

"3d. The glass does not act by extracting the poison, as may be seen from the experiments with strychnine.

"4th. The ligature, and the glass not exhausted, are equally as effectual, in cases of poisoned wounds, as the exhausted glass."

If cupping glasses and pressure do not extract the poison from wounds (and the latter of course does not) when they preserve the constitution from harm, what becomes of the deleterious substance; and how does it happen, that after an hour, or some other brief period, as in the experiments of Dr. R. the apparatus may be removed and the animal remain unaffected? Can strichnia be decomposed or assimilated, in such a situation, within that period? It would, we think, be difficult to sustain the affirmative of this proposition; and if this be correct, how does it happen, that after the pressure is withdrawn, the general system does not become affected? On this subject more light is wanted.

Under the analectic head of this department of our Journal, will be found an article from the 1st No. of the *London Medical and Surgical Journal*, on the influence of cupping glasses on the developement of the vaccine vesicle, to which we beg leave to recall the attention of the reader in connexion with what he has just read.

In the course of his inquiries, Dr. Rodrigue, three times applied hydrocyanic acid to the trunks of nerves, laid bare, and found that it produced no effect, except in one instance and then in a slight degree, and could be explained by the adhesion to the nerve of a small quantity of cellular membrane. From these experiments the Dr. "judges that prussic acid has no direct specific action on the nerves," and that it "does not act upon the system through the medium of the nerves." Now this may *possibly* be the case, but the experiments cited are, it appears to us, far from showing it; for, first, the poison was applied to the *neurilema* and not the *nervous matter*, of which alone, sound physiology predicates the faculty of transmitting impressions; and, secondly, it was not applied to the sensible or susceptible extremities, as found in the wounded skin or muscles, or in the healthy surface of one of the membranes, as that of the mouth or eye. That *mechanical* agents, directed upon the *trunks* of nerves, may greatly disturb the economy of the whole system we all know; but, for medicines or poisons, which act on a different principle, to affect the system, through the medium of the nerves, it would seem to be requisite to bring them in contact with their gensient extremities either external or internal. On this subject, we mean the mode in which prussic acid destroys life, there have been facts extant, ever since the day when Gay-Lussac, and Thenard, first presented to the world that, which for the good of mankind they might, perhaps, as well have let alone; and Dr. R. has added to the number, if not the variety of our *data*. Thus he found that a single drop of the acid, introduced into a wound in the thigh of a rabbit, produced violent convulsions in ten seconds, and perfect death in sixty; and that a similar application destroyed another in ten seconds; while a full grown cat, (not easily killed by common means,) expired in a minute, from the application to its *tongue* of a single drop of the poison! This is like the fall of the electrometer, when the charged battery is touched with a conductor:—we refer, of course, merely to the celerity of the operation in the two cases. For ourselves we require no other evidence than these and other facts of the same kind already known, to convince us, that prussic acid *does* exert on the nervous system, when applied to susceptible and unprotected surfaces, a most specific and deadly influence; and that through the medium of this system, it extinguishes life with a promptness and certainty, that ought to recommen it to the law givers of every nation, which seeks, in its capital punishments, the death only, and not the the torment of its criminals? This however by the way. As to the *quomodo* when all the functions cease in sixty seconds from the application of a single drop of prussic acid, we confess our utter ignorance: but that the entire nervous function is overthrown by the local impression, we cannot entertain a doubt.

